# Evaluation of a strain Long human respiratory syncytial virus with *M2–2* gene deletion in intranasally vaccinated BALB/c mice

**DOI:** 10.3389/fimmu.2026.1829916

**Published:** 2026-06-08

**Authors:** Ri-gan Shu, Jie Jun, Yun-xuan Zheng, Dan-ning Yue, Hao Hu, Hua-wei Xu, Xiao-lei Zhao, Hong-ru Wang, Xi-man Liu, Yi-peng Zhang, He Wang, Jie-mei Yu, Yan-peng Zheng, Xiang-lei Peng, Yuan-hui Fu, Jin-sheng He

**Affiliations:** College of Life Sciences and Bioengineering, Beijing Jiaotong University, Beijing, China

**Keywords:** human respiratory syncytial virus, immune efficacy, live-attenuated vaccine, *M2-2* gene, Subgroup A strain Long

## Abstract

**Background:**

Human respiratory syncytial virus (RSV) is a leading cause of lower respiratory tract illness (LRTI) worldwide, particularly in infants, the elderly and immunocompromised individuals. Despite this substantial disease burden, no licensed pediatric vaccines are currently available for infants aged ≥ 6 months, highlighting an urgent need for effective immunization strategies.

**Methods:**

Live-attenuated vaccines (LAVs) were developed and characterized based on RSV subgroup A strain Long (*wt*RSV) by deleting the *M2-2* gene alone or by further introducing silent mutations into the small hydrophobic (*SH*) gene alongside a 112-nucleotide deletion within its downstream untranslated region. Subsequently, the *in vitro* and *in vivo* characteristics of these constructed recombinant RSV strains were evaluated.

**Results:**

In HEp-2 and Vero cells, the recombinant strains RLΔM2-2 and RLΔM2-2112 displayed reduced replication capability compared with the parental *wt*RSV. In BALB/c mice, RLΔM2-2-infected groups demonstrated significantly lower lung viral loads and less pronounced body weight loss than those infected with RLΔM2-2112. Following intranasal immunization, RLΔM2-2 robustly induced systemic and pulmonary humoral and cellular immune responses including preF-specific antibodies, neutralizing antibody responses, and RSV-specific CD8^+^ T-cell responses characterized by a Th1-biased immune profile. Furthermore, the vaccine conferred protection against subsequent RSV challenge with no signs of enhanced respiratory disease (ERD).

**Conclusion:**

These results support the application of *M2-2* deletion strategy to the parental RSV strain Long backbone, providing a solid foundation for further evaluation and development of RSV LAVs.

## Introduction

The human respiratory syncytial virus (RSV) is the leading viral pathogen responsible for lower respiratory tract illness (LRTI) in populations of infants, young children, the elderly, and immunocompromised individuals worldwide. It has been estimated that children under 5 years of age who suffer from LRTI amount to 33 million cases, resulting in approximately 3.6 million hospitalizations and over 100,000 deaths per year globally (1). The risk of RSV infection is age-dependent, with nearly all children infected at least once by 2–3 years of age (2, 3). RSV was isolated in 1956 from chimpanzees and in 1957 from humans (4–6). The history of RSV vaccine development suffered a catastrophic setback in the 1960s due to the occurrence of vaccine-associated enhanced respiratory disease (ERD) in infants immunized with the pioneering formalin-inactivated whole-virus (FI-RSV) vaccine. These adverse events severely impede the development of RSV vaccine, and no vaccines are accessible for a long time. Recently, advancements in understanding the prefusion conformation of the fusion glycoprotein (F), one of the main neutralizing antigens of RSV, and in novel vaccine technologies have greatly facilitated the development of the RSV vaccine. In 2023 and 2024, three vaccines—Arexvy (GSK), Abrysvo (Pfizer), and mRESVIA (Moderna)—have been sequentially licensed by the U.S. Food and Drug Administration (FDA) for preventing RSV disease in individuals aged 60 and above, as well as individuals 18 through 59 years of age who are at increased risk for LRTI. Currently, the CDC recommends RSV vaccination for everyone 75 and over, and for adults ages 50–74 with risk factors.

Additionally, in order to protect young infants less than 6 months of age against severe RSV disease through maternal antibodies, the FDA also authorized the use of Abrysvo for pregnant individuals at 32 to 36 weeks′ gestation. Meanwhile, there are two kinds of long-acting monoclonal antibodies (mAbs), nirsevimab and clesrovimab, available for all infants under 8 months, and one of them, nirsevimab, is available for high-risk infants aged 8–19 months to prevent RSV diseases. However, there are no available RSV vaccines for vulnerable populations such as infants and young children aged 6 months to 5 years old.

Despite the remarkable achievements on RSV vaccines, pediatric RSV vaccines have been developed with great caution due to ERD. For instance, certain vaccine formulations, such as preF subunit vaccines, are generally avoided in RSV-naïve infants due to concerns that they might prime for ERD, a phenomenon previously documented in murine models (7). Another example is the recent temporary halt in December 2024 by the FDA in the administration of some vaccine candidates targeting RSV-naïve children because of the result of a phase 1 trial of two experimental Moderna RSV vaccines in infants aged 5 to 8 months. There were five cases of severe to very severe LRTIs reported among the 40 babies who received the vaccine dose, compared with just one case in the placebo group of 20 babies. Five infants required hospitalization, with one needing mechanical ventilation. Therefore, the FDA put a hold on trials concerning the pediatric development of other non-live-attenuated RSV vaccines (e.g., live-attenuated chimeric respiratory viruses, other viral vectored vaccines, mRNA, and recombinant particle/subunit vaccine candidates) (8, 9). In contrast, RSV live-attenuated vaccines (LAVs) do not prime for ERD in RSV-naïve infants and currently are the only acceptable approach to the development of pediatric RSV vaccines (8, 10). Since 1997, thousands of newborns and RSV-seronegative children have participated in trials evaluating RSV LAV candidates, but no cases of ERD are observed (10–25). Until now, at least five vaccine candidates have exhibited an efficacy of 67% (95% confidence interval [CI], 24 to 85) against medically attended RSV acute-respiratory illness (RSV-MAARI) and 88% (95% CI, −9 to 99) against medically attended RSV lower respiratory illness (RSV-MAALRLI) (11, 26). One of them, RSV/ΔNS2/Δ1313/I1314L or RSVt, demonstrated promising immunogenicity profiles among infants and toddlers, without identified safety concern based on results of phase 1/2 clinical trials (27) and entered a phase 3 clinical trial (NCT06252285) in 2024 with approximately 6,300 6- to 22-month-old children enrolled across 19 countries. Regrettably, because of the unmet efficacy, this RSV candidate was terminated in October 2025. However, the safety profile was acceptable, and no signals of ERD were observed (28). These data indicated that RSV LAVs have a great safety profile in RSV-naïve children, but immunogenicity still requires enhancement.

Besides the fact that RSV LAVs are constructed based on the deletion of the *NS2* gene, another family of RSV LAV candidates are constructed from the deletion of the *M2–2* gene, regulating viral RNA genome synthesis. The strategy exhibited diminished genome replication but enhanced gene transcription and protein expression, thereby conferring full protection against RSV challenge in mice (29–31). Compared with RSVt at 10^6^ PFU dose during phase 1 clinical trials, some candidates of the *M2-2*-deleted RSV LAV family such as MEDIΔM2-2, RSV276, and LID/ΔM2-2/1030s demonstrated a similar safety profile and an enhanced immune efficacy in RSV-seronegative children at 10^5^ PFU dose (11, 12, 19–21, 23). Interestingly, compared to MEDIΔM2-2, all other candidate strains within this family exhibited differential attenuation phenotypes to some extent; for instance, LIDΔM2-2—which was constructed based on a different A2 strain (GenBank accession number KT992094) and differs in its genetic modifications—displayed an insufficiently attenuated phenotype (19, 23).

To date, most RSV LAV candidates are derived from the parent A2 strain; although they have made significant clinical progress, no products are licensed yet. Therefore, exploring the alternative parent strains including strain Long is a rational choice. Many studies have observed phenotypic differences between RSV strains *in vitro* and in animal models, although the molecular bases for these strain-specific phenotypes are largely unknown (32). For example, the RSV strain Long is classified in the same subgroup A and GA1 genotype as the A2 strain, owing to its high degree of nucleotide identity with the A2 strain (e.g., 98% for the F protein and 96% for the G protein); it also elicits identical Th1-biased CD8^+^ CTL responses. However, strain Long can trigger significantly higher IFN-α levels in human dendritic cells, suggesting a potentially different immunological profile from the A2 strain (33–36). Furthermore, consistent with the aforementioned strategies, targeting the nonessential accessory protein M2-2 represents a promising approach to developing RSV LAVs with enhanced profiles of both attenuation and immunogenicity. These data demonstrated that the extent of attenuation varied among RSV LAVs generated from the deletion of *M2–2* ORF. This variation seemed dependent both on specific sequences within the MEDI/ΔM2-2 A2 background that are missing or altered in other backgrounds (LID/ΔM2-2), and on the alternative design of *M2–2* deletion itself (19, 20, 37, 38), although its underlying mechanisms remain unclear. Additionally, we previously generated RSV LAV candidates based on strain Long by introducing single or combined A2-derived attenuation mutation—specifically cold-passaged (*cp*), temperature-sensitive (*ts*), and the small hydrophobic (*SH*) gene deletion (ΔSH) modifications. Notably, these Long-based candidates recapitulated attenuation phenotypes similar to their A2-derived counterparts both *in vitro* and in a murine model (39). Altogether, given the potential difference in immune phenotypes between strains A2 and Long, and the fact that attenuation phenotype of *M2-2*-deleted RSV LAV candidates can be shaped by both the viral genetic backbone and the specific design of the *M2–2* deletion, the RSV strain Long represents a promising parental backbone for alternative LAV development. Therefore, we sought to evaluate whether the *M2–2* deletion strategy could be applied to the RSV strain Long background to generate RSV LAV candidates with balanced attenuation and immunogenicity.

In the present study, we developed two recombinant RSVs, designated RLΔM2–2 and RLΔM2-2112. Both were derived from the RSV strain Long, each harboring a deletion of 207 nucleotides (nt) within the *M2–2* ORF. In addition to the M2-2 deletion, RLΔM2-2112 was further engineered to contain silent mutations in *SH* gene alongside a 112-nt region deletion in the downstream untranslated region (UTR) of the *SH* gene. Following the recovery of these two RSV vaccine candidates through reverse genetics, we assessed the attenuation phenotypes of RLΔM2–2 and RLΔM2-2112 by measuring their replication activity both *in vitro* and *in vivo*. Subsequently, BALB/c mice were intranasally (i.n.) inoculated with a single dose of the more attenuated RLΔM2–2, and were later challenged with parental wild-type (*wt*) RSV strain Long (*wt*RSV). The immunogenicity and protective efficacy of this vaccine candidate were comprehensively evaluated in the immunized and challenged mice. This evaluation was achieved by analyzing systemic and pulmonary humoral and cellular immune responses and neutralizing antibody titers, as well as viral replication levels and lung histopathology in the primed and challenged mice.

## Materials and methods

### Cells and viruses

HEp-2 and Vero cells (ATCC, Rockefeller, MD, USA) were cultured in Dulbecco’s Modified Eagle Medium (DMEM, HyClone, Logan, UT, USA), supplemented with L-glutamine (2 mmol/L, Gibco, Grand Island, NY, USA), antibiotics (40 IU/mL penicillin G and 100 μg/mL streptomycin, Eallbio, Beijing, China), and 10% fetal bovine serum (FBS, HyClone). *wt*RSV (ATCC) was propagated using HEp-2 cells maintained in DMEM containing 2% FBS, L-glutamine, and antibiotics.

### Rescue and plaque purification of recombinant RSVs

**Figure 1:**
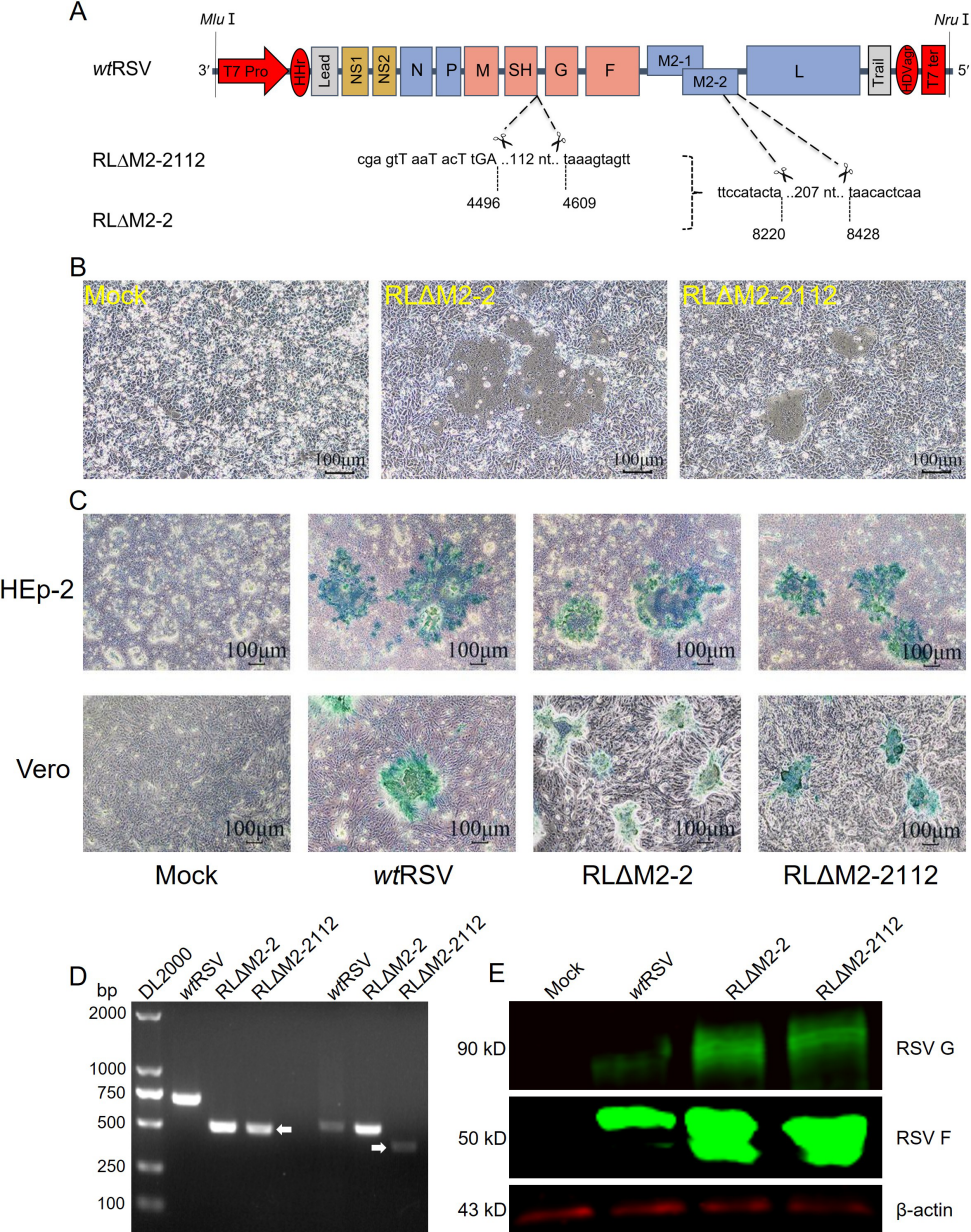
Virus *in vitro* characteristics. **(A)** Schematic diagram of the antigenome structure of recombinant RSVs and their control elements. **(B)** Cytopathic effects (CPE). Typical RSV syncytia were clearly visible under the microscope (Ts2R-FL, Nikon, Japan) at 48 h post-infection of HEp-2 cells with 0.01 MOI virus. Mock, uninfected HEp-2 cells. **(C)** Immunoplaque analysis of HEp-2 and Vero cells infected with serially diluted virus samples of either *wt*RSV, RLΔM2-2, or RLΔM2-2112. For the immunoplaque analyses, the primary antibody was a goat anti-RSV polyclonal antibody (AB1128, Millipore, Billerica, MA, USA). Mock, uninfected HEp-2 cells or Vero cells. **(D)** Agarose gel electrophoresis analysis of RT-PCR products. Vero cells were infected with *wt*RSV, RLΔM2-2, or RLΔM2-2112, and viral RNA was extracted for RT-PCR analysis. Lanes 2-4 were amplified using primers spanning the *M2–2* deletion region, whereas lanes 6-8 were amplified using primers spanning the *SH* downstream deletion region. The left and right arrows indicate the PCR products corresponding to the partial deletion of the *M2–2* gene and the 112-nt deletion downstream of the *SH* gene, respectively. **(E)** Western blot analysis of the expression levels of G and F proteins in the infected HEp-2 cells. The primary antibodies against G and F proteins were goat anti-human RSV polyclonal antibody (AB1128, Millipore) and rabbit anti-human RSV-F monoclonal antibody (11049-R302, Sino Biological, Beijing, China), respectively. The fluorescence intensity was analyzed using a dual-color near-infrared fluorescence imaging system. Mock, uninfected HEp-2 cells. The expression levels of β-actin were used as internal reference. HHr, hammerhead ribozyme; T7 Pro, T7 promoter; Lead, leader; Trail, trailer; HDVagr, HDV antigenomic ribozyme; T7ter, T7 terminator; *wt*RSV, RSV strain Long.

The plasmid pRLΔM112 was constructed to harbor a cDNA encoding antigenome of the parental *wt*RSV (GenBank accession number AY911262), with a 207-nt region deleted from the *M2–2* ORF (nt 8221–8427 relative to GenBank AY911262). In addition, a 112-nt region within the downstream UTR of the *SH* gene (nt 4497–4608 relative to GenBank AY911262) was deleted and five silent nt changes were introduced into the 3′ end of the *SH* ORF (4487C > T, 4490C > T, 4493A > T, 4495A > G, 4496G > A). The entire antigenomic cDNA was flanked by regulatory elements including the T7 promoter, T7 terminator, and ribozymes (**Figure 1A**), as described previously (39). The plasmid pRLΔM2-2, similar to pRLΔM112 deleting the *M2–2* gene but with the intact *SH* gene as MEDIΔM2–2 and RSV/276, was constructed in a bacterial artificial chromosome (BAC) from pSmartBAC (Lucigen, Middleton, WI, USA). The sequences of the resulting constructs were confirmed by whole plasmid sequencing (Tsingke, Beijing, China). For the rescue of recombinant RSVs, HEp-2 cells were infected with MVA-T7, kindly provided by Prof. Bernard Moss (NIH, MD, USA), at a multiplicity of infection (MOI) of 3 and co-transfected with the plasmid containing the full-length anti-genomic cDNA and four helper plasmids [pCITE-N, pCITE-P, pCITE-L, and pCITE-M2-1, kindly provided by Dr. Marie-Anne Rameix-Welti (Unite´ de Virologie et Immunologie Moléculaires, Université Paris-Saclay, Paris, France)] using Lipofectamine 2000 (Invitrogen, Carlsbad, CA, USA) according to the manufacturer’s instructions. The cells were then incubated for 3–5 days at 37°C in a 5% CO_2_ incubator.

The harvested cell suspension was subjected to plaque purification in HEp-2 cells (40). Briefly, cells were seeded in six-well plates and infected with serially diluted virus samples. After 1 h of adsorption at 37 °C, the inoculum was removed, and cells were overlaid with 2% agarose in 2×DMEM supplemented with 4% FBS. The plates were incubated at 37°C with 5% CO_2_ for 4–7 days until distinct plaques formed or the cytopathic effects (CPEs) were observed. Individual plaques were picked using a sterile pipette tip and transferred into DMEM. The virus was released by freeze–thaw cycles and used for further amplification in fresh HEp-2 cells. The plaque purification process was repeated at least twice to ensure clonal purity. Then, the purified viruses were passaged to Vero cells in DMEM supplemented with 2% FBS at 37°C, L-glutamine (2 mmol/L), and antibiotics (40 IU/mL penicillin G and 100 μg/mL streptomycin). After obvious CPEs were observed, the viruses were harvested and subjected to the subsequent process (39, 41).

### Virus preparation

RLΔM2–2 or RLΔM2–2112 was purified using sucrose (Sigma-Aldrich, St. Louis, MO, USA) density gradient ultracentrifugation (42). Briefly, virus-containing supernatants were clarified by centrifugation at 3,000 rpm for 15 min at 4 °C, then the cell pellet and the supernatant were separately collected. For the cell pellet, it was resuspended with 5–10 mL of phosphate-buffered saline (PBS), and the resulting suspension was transferred to a pre-cooled 50-mL centrifuge tube, then frozen and thawed twice at 37 °C and −80 °C, and centrifuged at 3,000 rpm and 4 °C for 15 min to remove the cell debris and retain the supernatant. The clarified supernatant was combined and mixed with 50% (W/V) polyethylene glycol (PEG) 6000 solution at a 10% (V/V) volume ratio and gently stirred at 4 °C for 90 min using a magnetic stirrer. The virus was then pelleted by centrifugation at 5,000 rpm, 4 °C for 20 min, and the supernatant was discarded. The viral pellet was resuspended in pre-cooled NTE buffer (NT buffer containing 0.1 M MgSO_4_ and 1 mM EDTA) at 1%–5% of the original volume. The virus suspension was layered onto a discontinuous sucrose gradient (30%–45%–60%) and centrifuged with P40ST rotor (Hitachi, Tokyo, Japan) at 35,000 rpm for 90 min at 4 °C. The viral band located between the 30% and 45% sucrose layers was carefully collected and diluted in NT buffer (containing 0.1 M MgSO_4_). The diluted virus suspension was further pelleted by ultracentrifugation with P55ST2 rotor (Hitachi) at 22,000 rpm, 4 °C for 90 min, and the final viral pellet was resuspended in NTE buffer, aliquoted, and stored at −80 °C for subsequent experiments.

FI-RSV was prepared following a previously described protocol with minor modifications (42, 43). Briefly, RSV-infected cells were harvested and subjected to three cycles of rapid freezing in liquid nitrogen and thawing at 37°C to release intracellular virus particles. The resulting lysates were then clarified by centrifugation at 550 g for 15 min at 4 °C to remove large debris. The clarified lysates were then incubated with 37% formalin (Sigma-Aldrich) at a final dilution of 1:4,000 (V/V) for 3 days at 36 °C with gentle mixing. Following inactivation, the virus was pelleted by ultracentrifugation at 17,000 rpm (P28S rotor) for 1 h at 4 °C. The resulting viral pellet was resuspended in serum-free DMEM at 1/25 of the original volume. The total protein concentration of the inactivated virus was determined using the BCA protein assay kit (Thermo Fisher Scientific, Waltham, MA, USA). Notably, the content (mg) of FI-RSV that corresponds to 5.0 × 10_5_ PFU of live virus prior to formalin inactivation will be determined and taken to inoculate the mice intramuscularly (i.m.) based on the calculations.

### Immunoplaque assay

The serially diluted RSV samples were inoculated to 90% confluence of Vero cells in a 96-well plate in triplicate for 1 h at 37°C. Subsequently, the samples were discarded and the cells were rinsed twice with 1× PBS. Then, DMEM containing 0.75% methyl cellulose (Sigma-Aldrich) was added to the wells. After 3 days of incubation at 37°C under 5% CO_2_, the cell monolayer was fixed with 95% cold alcohol, and viral replication activity could be assessed using a goat anti-RSV polyclonal antibody (AB1128, Millipore, Billerica, MA, USA) incubated with horseradish peroxidase rabbit anti-goat IgG (Santa Cruz Biotechnology, Santa Cruz, CA, USA), and visualized after adding TMB (Promega, Madison, WI, USA) under the microscope (Nikon). RSV titers were expressed as plaque-forming units per milliliter (PFU/mL) (39).

### The construction, rescue, and identification of the recombinant RSVs genetically, morphologically, and immunologically *in vitro*

RLΔM2–2 and RLΔM2–2112 were constructed and designed by deleting the same encoding gene of *M2–2* but different modification of the RSV genome. To identify all the modifications on the genomes of the recombinant RSVs, the RT-PCR method was employed. The viral RNA samples were extracted from RSV-infected cells with an RNA extraction kit (Yeasen, Shanghai, China) according to the manufacturer′s instructions. Viral RNA was reverse transcribed, and a cDNA fragment spanning the M2 gene was amplified with primer M2-2-F1 (5′-TGCTAGAGAGTTATATAGGATC-3′) and primer M2-2-R1 (5′-TCGGTTAGATAAACATTAGCAG-3′) for detecting *M2–2* deletion, and with primer 112-F1 (5′-CTCCATCATGACTGCAATAC-3′) and primer 112-R1 (5′-GGTTGTGTTCTTGATCTGGC-3′) for detecting *SH* gene deletion. The resultant DNA products from the RT-PCR were analyzed on a 1.2% agarose gel and visualized by nucleic acid staining. For the *M2–2* gene deletion analysis, the anticipated sizes of DNA products are 497 base pairs (bp) from M2-2-deleted recombinant RSVs and 704 bp from *wt*RSV, respectively. For the *SH* gene deletion analysis, the resulting sizes of DNA products are 433 bp from RLΔM2-2112 and 545 bp from RLΔM2–2 and RSV Long, respectively.

To further analyze the full-length genomes of the rescued viruses, the whole genomes of RLΔM2–2 or RLΔM2–2112 were subjected to deep sequencing. Total RNA was extracted from virus-infected cells using the RNA extraction kit (Yeasen) following the manufacturer’s instructions. RNA quality and integrity were assessed using an agarose gel electrophoresis. Total RNA concentration was measured using the Qubit™ 2.0 RNA Assay Kit (Invitrogen, Carlsbad, CA, USA). Ribosomal RNA was depleted using RiboMinus™ (Thermo Fisher Scientific), and cDNA libraries were prepared with Superscript IV Reverse Transcriptase (Invitrogen) and the NEBNext Ultra II RNA Library Prep Kit (New England Biolabs, Ipswich, MA, USA). The libraries were quantified using Qubit and Agilent 2100 Bioanalyzer (Agilent Technologies, Santa Clara, CA, USA) before sequencing on the Illumina NovaSeq 6000 platform (paired-end 150-bp reads). Raw sequencing reads were quality-checked using FastQC, trimmed with Trimmomatic, and mapped to the RSV reference genome using BWA or Bowtie2. Variant calling was performed with GATK’s HaplotypeCaller, and functional annotation was conducted using SnpEff. Sequencing depth and coverage were analyzed using BEDTools to ensure data quality.

Infected cells with either RLΔM2–2 or RLΔM2–2112 will express different kinds of proteins such as F protein to accomplish the replication cycle. Therefore, the expressed proteins were assayed by Western blot, and HEp-2 cells were infected with 5 MOI of the recombinant RSVs and *wt*RSV, respectively, collected 33 h post-infection, washed with cold PBS, and lysed in RIPA buffer. Proteins were separated under reducing conditions (100 mmol/L 2-mercaptoethanol, boiled) on 10% SDS-PAGE. After transferring to nitrocellulose membranes, proteins were incubated with goat anti-human RSV polyclonal antibody (Millipore), rabbit anti-human RSV-F mAb (Sino Biological, Beijing, China), IRDye 800CW labeled donkey anti-goat IgG (LI-COR, Lincoln, NE, USA), and goat anti-rabbit IgG (LI-COR). Visualization was performed using an infrared fluorescence imaging system (LI-COR). Lysates of uninfected HEp-2 cells and cells infected with *wt*RSV served as negative and positive controls, respectively.

RSV virions appear as heterogeneous particles with different sizes and shapes, among which the long filamentous forms often predominate. For observation of the virus morphology, the purified RSVs obtained through sucrose gradient centrifugation, as mentioned above, were negative-stained with 1% phosphotungstic acid to prepare samples for transmission electron microscopy (TEM; JEM-1400, JEOL, Japan). To examine the budding process of RSV, RSV was inoculated into HEp-2 cells at 5 MOI. After 20 h, cells were collected and fixed sequentially with 2.5% glutaraldehyde and 1% osmium tetroxide. Following resin embedding, ultrathin sections were prepared and stained with 2% uranyl acetate and 0.2% lead citrate for observation under TEM. The samples for immunoelectron microscopy (IEM) were treated sequentially by using a specific rabbit anti-human RSV F protein mAb (11049-R302, Sino Biological, Beijing, China) and goat anti-rabbit IgG labeled by colloidal-gold (10 nm, K1034G-G10, Solarbio, Beijing, China), then prepared as mentioned above before they were observed under TEM.

### The growth kinetics of recombinant RSVs *in vitro*

The growth kinetics of RLΔM2–2 or RLΔM2–2112 were assessed using a previously described method (29). HEp-2 and Vero cells were infected with either *wt*RSV, RLΔM2-2, or RLΔM2–2112 at 0.01 MOI in triplicate and incubated at 37°C in a 5% CO_2_ incubator. Cells and the supernatants were harvested together at 24-h intervals post-infection, and viral titers were determined by immunoplaque assay, as introduced above.

### The replication levels of recombinant RSVs in the lung of the infected mice

BALB/c mice aged 7–8 weeks were obtained from Charles River Laboratories, Beijing, China. This study was carried out in accordance with the recommendations in the Guide for the Care and Use of Laboratory Animals of the People’s Republic of China. Mice were anesthetized with 2.5% Avertin (tribromoethanol) at a dosage of 0.14 mL per 10 g of body weight administered intraperitoneally, which reliably induced unconsciousness during intranasal inoculation and other experimental manipulations. At the endpoint of the study, animals were euthanized by gradual-fill CO_2_ inhalation until respiratory arrest. This method was selected to provide a rapid and humane death while minimizing pain and distress.

To assess the replication and attenuation levels of the recombinant RSVs *in vivo*, BALB/c mice were divided into four groups (10 per group) randomly and infected i.n. with 5.0×10^5^ PFU of RLΔM2-2, RLΔM2-2112, *wt*RSV, or PBS in a volume of 50 μL under anesthesia. Five mice from each group were weighed daily for seven consecutive days. The other 5 mice in each group were sacrificed on day 4 post-infection, and the lungs were harvested, weighed, and homogenized using a glass tissue grinder. Lung viral loads were determined using quantitative real-time polymerase chain reaction (RT-qPCR) with specific primers for N gene (39). Briefly, 0.1 g of lung tissue was homogenized in 1 mL of lysis buffer, and lung viral RNA was then extracted using a total RNA extraction kit (Yeasen) according to the manufacturer′s instructions. Then, RT-qPCR was performed using a SYBR green probe (Tiangen Biotech, Beijing, China). The primers for the RSV N gene were as follows: forward primer, 5′-ACAAAGATCAACTTCTGTCATC-3′, and reverse primer, 5′-GCACATCATAATTAGGAGTATC-3′.

### The immunogenicity of RLΔM2–2 analyzed following the vaccination of mice

To assess the immunogenicity of RLΔM2–2 in BALB/c mice, BALB/c mice (Charles River Laboratories) were anesthetized with Avertin and inoculated i.n. with either 5.0×10^5^ PFU of RLΔM2-2, *wt*RSV, or PBS in a volume of 50 μL. On days 0 and 28, sera were collected from the orbital plexus of mice. On day 28, bronchoalveolar lavage (BAL) fluids and NW were collected according to the method reported previously (44). Titers of binding antibodies against preF in sera, BAL, and NW were detected by the enzyme-linked immunosorbent assay (ELISA) method. Briefly, ELISA plates (Nest, Wuxi, China) were coated with the purified preF (purified in our laboratory) overnight at 4 °C and blocked with 5% BSA in PBS for 2 h at 37 °C, and BAL, NW, or serially diluted sera were added to the plates. After the addition of horseradish peroxidase (HRP)-conjugated goat anti-mouse IgG, IgG1, or IgG2a (Santa Cruz), the wells were incubated with soluble TMB substrate (Biodragon, Suzhou, China) and detected at 450 nm using an ELISA plate reader (Agilent BioTek, Santa Clara, CA, USA).

Fluorescent-based neutralization assays (FNAs) were performed as previously described (44). Serum samples were heat-inactivated at 56 °C for 30 min. Serially diluted sera were mixed with rRSV-EGFP (41) and incubated for 1 h at 37 °C, then the mixtures were added to 96-well plates with HEp-2 cells. After incubation for 48 h at 37 °C, the fluorescence intensity was detected using a microplate reader (Molecular Devices, San Jose, CA, USA) with an excitation wavelength of 479 nm and emission wavelength of 517 nm. Neutralizing antibody titers were calculated as the antibody concentration caused a 50% reduction in fluorescence intensity.

A mouse interferon-gamma (IFN-γ) precoated enzyme-linked immunospot (ELISpot) assay kit (Dakewe, Beijing, China) was utilized to assess the M2-1-specific CD8^+^ T-cell response, following the manufacturer’s instructions. Briefly, lymphocytes were collected from the spleen and pulmonary mediastinal lymph nodes (PMLNs) and stimulated with H-2K^d^-restricted cytotoxic T lymphocyte (CTL) epitope polypeptides (SYIGSINNI and VYNTVISYI) from the RSV M2–1 protein (45) (synthesized by Sangon Biotech, Shanghai, China) for 24 h. The frequency of responding cells was quantified using an ELISpot reader (BD Biosciences, Franklin Lakes, NJ, USA).

### The protective efficacy of RLΔM2-2-vaccination against *wt*RSV challenge in mice

To evaluate the immune efficacy of RLΔM2–2 against RSV challenge, BALB/c mice were randomly grouped and inoculated i.n. with either 5.0×10^5^ PFU of RLΔM2-2, *wt*RSV, or PBS in a volume of 50 μL, or intramuscularly (i.m.) with 50 μL of FI-RSV (5.0×10^5^ PFU). On day 28 post-inoculation, these mice were challenged i.n. with 10^6^ PFU of *wt*RSV. Four days following the challenge, the mice were euthanized, and the right lungs were harvested for viral titration via RT-qPCR as described above. Briefly, the right lungs were weighed, placed in 1×PBS containing 1% bovine serum albumin, and homogenized with a glass tissue grinder. Viral RNA was then extracted using a total RNA extraction kit (Yeasen) following the manufacturer′s instructions. To assess the underlying risks of the vaccine to induce enhanced histopathological changes post-RSV challenge, the left lungs were fixed in 10% formalin in 1× PBS and subsequently embedded in paraffin. Five-micron-thick sections were stained with hematoxylin and eosin (HE). All slides were evaluated by a board-certified pathologist for thickening of alveolar septa, interstitial pneumonia, and perivascular inflammatory cell infiltration using a semiquantitative scale ranging from 0 to 5 (0 = absent; 5 = maximum/severe).

### Statistical analysis

Statistical analysis was performed with SPSS21 software (SPSS, Chicago, IL, USA), and the means of multiple groups were compared by one-way analysis of variance. *p* < 0.05 was considered significant.

## Results

### Virus rescue and characteristics identified *in vitro*

Through eliminating the function of the *M2–2* gene, we developed two recombinant RSVs based on *wt*RSV by deleting the 207-nt region of the *M2–2* ORF either alone or together with the 112-nt region from the downstream UTR of the *SH* gene and the modification of the last four codons. These recombinant RSVs were designated RLΔM2–2 and RLΔM2-2112, respectively. The deletion of the downstream UTR of the *SH* gene and the modification of the last four codons (**Figure 1A**) enhanced the stability of RSV antigenomic cDNA in bacterial hosts and facilitated plasmid cloning in the studies by our group and others (38, 39, 41). The RLΔM2–2 and RLΔM2–2112 were recovered following previously established methods (39, 41) and subsequently subjected to two rounds of plaque purification and the serially passage in Vero cells. Both recombinant viruses exhibited typical CPE, characterized by syncytium formation (**Figure 1B**) and displayed a plaque morphology similar to that of *wt*RSV following infection of HEp-2 and Vero cells (**Figure 1C**).

To confirm that the recovered viruses contained the corresponding deletions in the *M2–2* and *SH* genes, viral RNA was extracted from the infected Vero cells and subjected to RT-PCR with two primer pairs spanning the *M2* and the *SH* gene, respectively. After the RT-PCR products were separated by agarose gel electrophoresis, *wt*RSV yielded a PCR product of the expected size of 704 bp, while the two recombinant viruses with the *M2–2* deletion produced shorter fragments of 497 bp, respectively. Similarly, using primers specific for the *SH* gene, RLΔM2–2112 generated a shorter PCR product of 433 bp compared with the corresponding products of 545 bp from *wt*RSV and RLΔM2-2 (**Figure 1D**). These results were further confirmed by DNA sequencing analyses (data not shown).

Whole-genome deep sequencing analysis revealed the anticipated deletions of *M2–2* gene and *SH* downstream gene, respectively, and the distinct mutation patterns in the progeny viruses of the two attenuated RSV strains. In RLΔM2-2, mutations were predominantly enriched in the L and P genes, whereas in RLΔM2-2112, the majority of mutations were concentrated in the G gene. Although genetic differences were observed between progeny and parental viruses at the nucleotide level, the overall number and extent of mutations were limited. Importantly, no nonsense mutations were detected. Most missense mutations led to amino acid substitutions with similar physicochemical properties. The differential distribution of mutations between the two vaccine candidates may indicate strain-specific evolutionary dynamics from recombinant viruses with or without the deletion of 112 nt, although the underlying mechanisms remain to be elucidated (**Supplementary S1**). All these data suggested that the progeny viruses retained a high degree of genetic stability.

The *in vitro* expression levels of the neutralizing antigens F and G proteins were assessed using Western blot. Under denaturing and boiling conditions, the approximately 50-kDa F1 fragment and 90-kDa G proteins were detected. Consistent with earlier research (29), two recombinant viruses with the *M2–2* gene deletion showed stronger F and G protein signals than *wt*RSV (**Figure 1E**). Notably, the appearance of multiple or broad bands in samples infected with RLΔM2–2 or RLΔM2–2112 may be attributed to the high-level F protein expression, as well as to heterogeneous or incomplete glycosylation occurring within the relatively short interval between infection and harvest.

**Figure 2:**
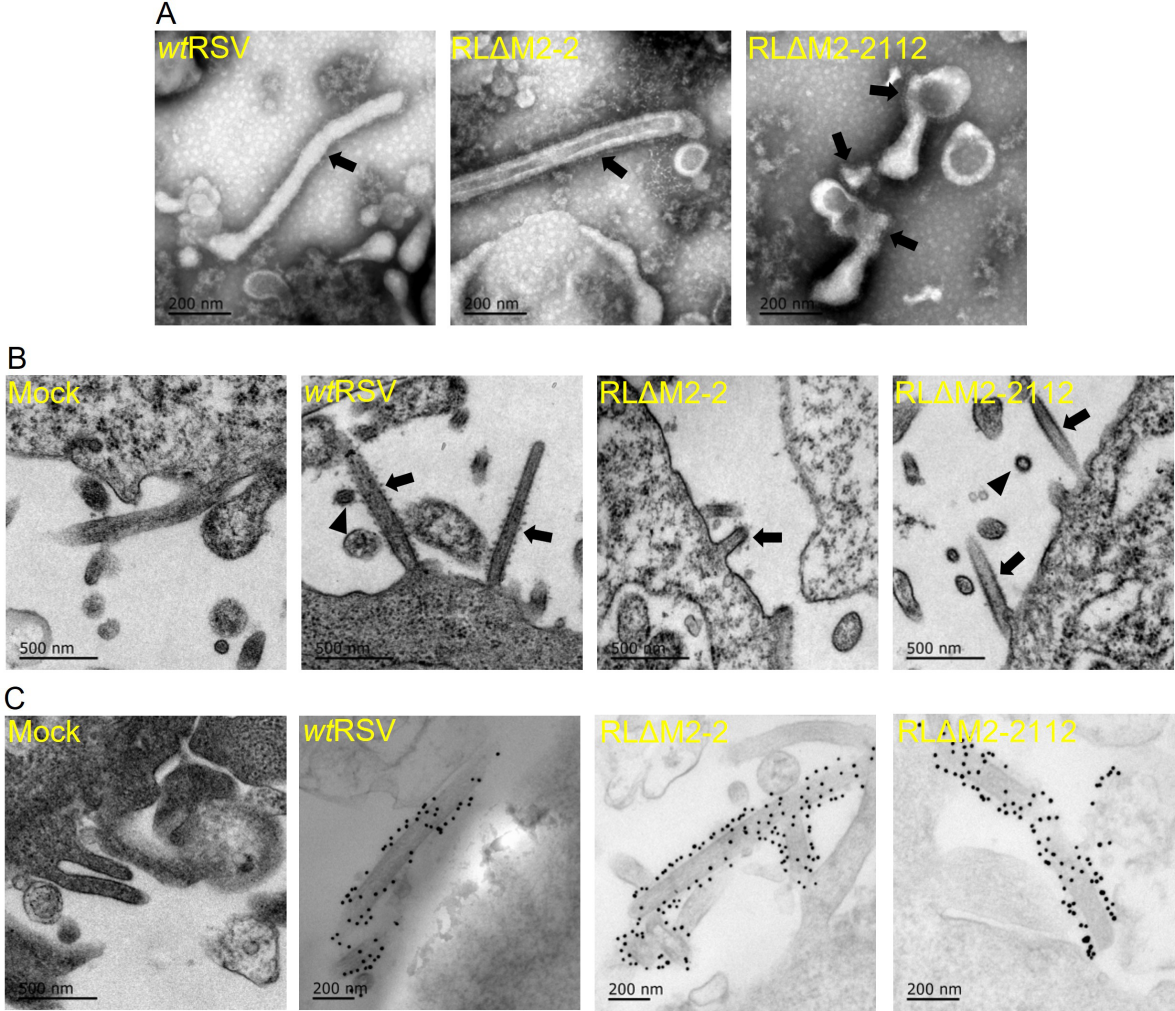
The morphology observed under transmission electron microscopy (TEM) and immunoelectron microscopy (IEM). **(A)** Purified viruses of *wt*RSV, RLΔM2-2, and RLΔM2–2112 were negatively stained with 1% phosphotungstic acid and observed using TEM (JEM-1400, JEOL, Japan). **(B)** Viruses were observed budding from the surface of the host cell membrane. The infected HEp-2 cells with the viruses were fixed, cut, stained, and observed under TEM. Mock: uninfected HEp-2 cells. **(C)** IEM analysis of rescued RSV particles. Samples were sequentially incubated with a rabbit monoclonal antibody against RSV F protein (11049-R302, Sino Biological, Beijing, China) and colloidal gold-labeled secondary antibodies (goat anti-rabbit IgG, 10 nm particle size, K1034G-G10, Solarbio, Beijing, China), then prepared as described in **(B)** Mock: uninfected HEp-2 cells. The arrow indicates budding filamentous virions, while the arrowhead indicates cross-sections of the virions. *wt*RSV, RSV strain Long.

To observe the morphology of the rescued viruses, we prepared TEM samples using purified viral particles. As expected, numerous filamentous particles and vesicular or irregular spherical structures were observed, all of which were enveloped, densely covered with spikes, and exhibited characteristically pleomorphic appearance (**Figure 2A**). RSV replicates in the cytoplasm and matures by budding from the host cell membrane. To investigate the budding process at the cell surface, HEp-2 cells were infected separately with the viruses. After 20 h, the cells were harvested for TEM analysis. The typical viral budding structures or their annular cross-sections, characterized by prominent surface spikes, were clearly observed on the host cell surface (**Figure 2B**). Additionally, the samples for IEM were prepared and assayed using a mAb against RSV F protein, followed by colloidal gold-conjugated secondary antibodies (46, 47), confirming that the observed surface spikes were indeed composed of viral F proteins. The specific binding of colloidal gold particles to the surface of virions further characterized these two rescued recombinant RSVs morphologically and immunologically (**Figure 2C**). These ultrastructural observations demonstrated that these recombinant RSVs shared identical morphological characteristics with *wt*RSV.

### The growth kinetics of recombinant RSVs

**Figure 3:**
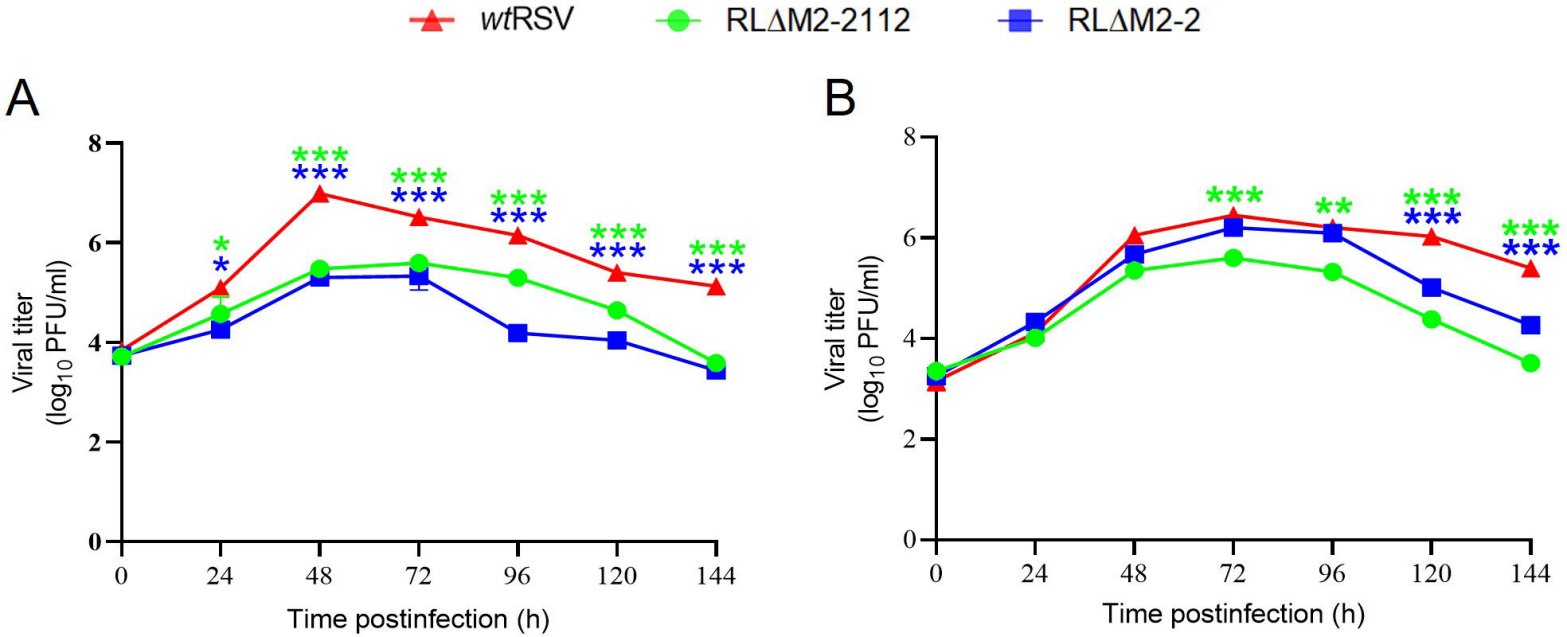
Multi-step growth curves. HEp-2 cells **(A)** and Vero cells **(B)** were grown in a 96-well plate and infected with RSV strain Long (*wt*RSV), RLΔM2-2, and RLΔM2–2112 at 0.01 MOI in triplicate, respectively. Cultures were harvested at 24-h intervals since 0 h following infection, and virus titers were determined in Vero cells using an immunoplaque assay at various time points. Data are presented as means ± standard deviation (SD), one representation of three repeated experiments. *p < 0.05, **p < 0.01, ***p < 0.001.

To compare the replication activity of these recombinant RSVs with the *wt*RSV, HEp-2 and Vero cells were inoculated with 0.01 MOI, and virus yields were monitored daily for 6 days using an immunoplaque assay. In HEp-2 cells, *wt*RSV peaked at 48 h, achieving a titer of 1.1×10^7^ PFU/mL, while RLΔM2–2 and RLΔM2-2112 peaked at 72 h with titers of 2.1×10^5^ and 3.9×10^5^ PFU/mL, respectively (**Figure 3A**). In Vero cells, *wt*RSV peaked at 72 h, reaching a titer of 2.8×10^6^ PFU/mL, whereas RLΔM2–2 and RLΔM2-2112 peaked at 72 h with titers of 1.6×10^6^ and 4.1×10^5^ PFU/mL, respectively (**Figure 3B**). These results indicated that in HEp-2 cells, viruses lacking the *M2–2* gene exhibited significantly lower replication capacity than *wt*RSV (*p* < 0.001); however, in Vero cells, the replication capacity of these viruses was highly similar to that of *wt*RSV—particularly the RLΔM2–2 strain. These results were consistent with the earlier report based on MEDIΔM2-2, the counterpart of RLΔM2–2 from the RSV A2 strain, where this kind cell-type-dependent replication was investigated and compared across different host-origin cell lines and suggested that these RSV LAVs with the deletion of the *M2–2* gene may be the host range mutant (30). However, in the case of RLΔM2-2112, its performance appeared to differ slightly from that of RLΔM2-2. The underlying mechanism remains unclear.

### *In vivo* attenuation and body weight changes in BALB/c mice following intranasal infection of recombinant RSVs

**Figure 4:**
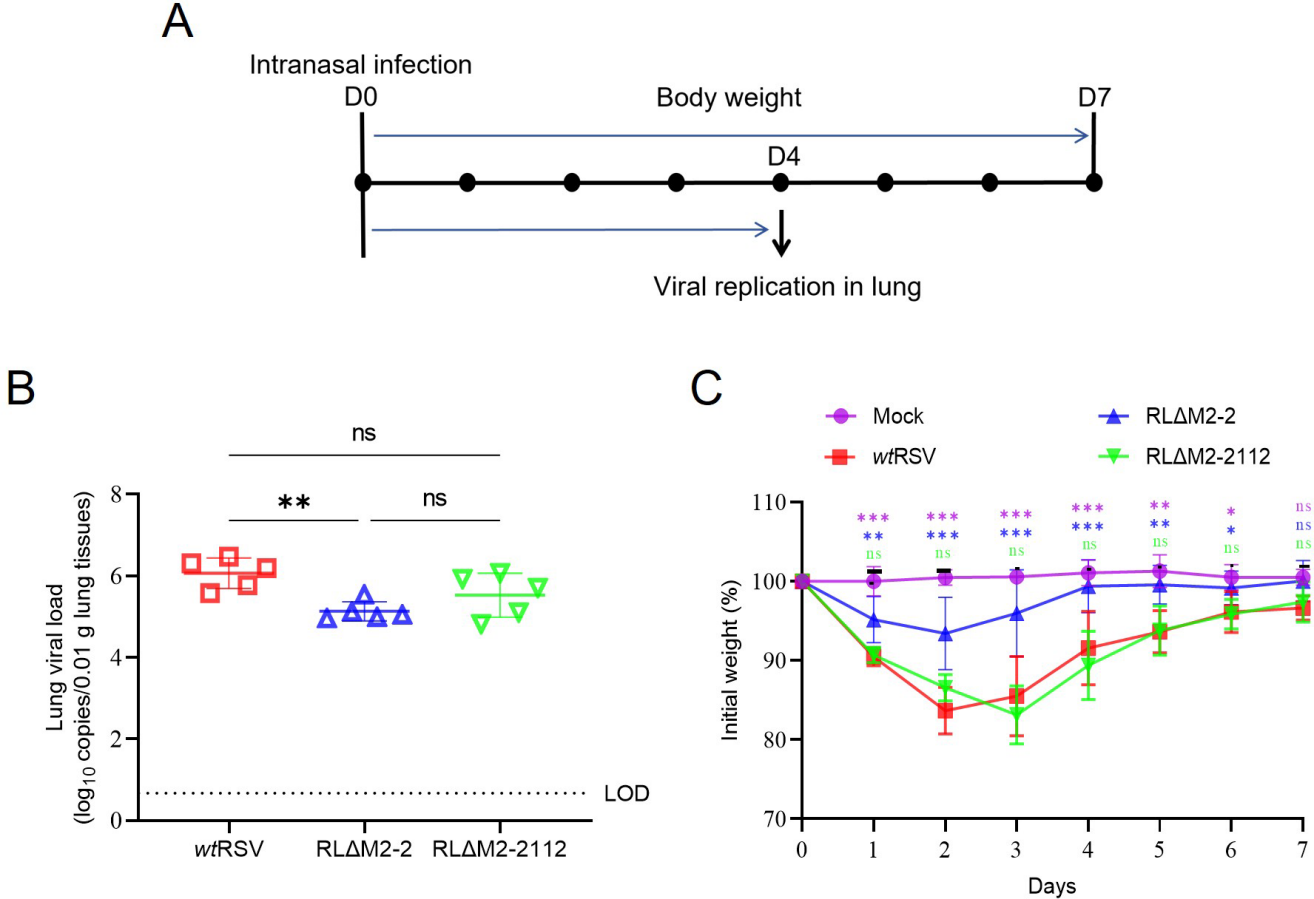
Evaluation of the viral replication in the lung and the resulting body weight loss in BALB/c mice following the intranasal infection of the recombinant viruses. Schematic diagram of the animal experiment **(A)**. BALB/c mice aged 7–8 weeks old were grouped 10 mice per group and infected with 5.0×10^5^ PFU of RLΔM2-2, RLΔM2-2112, or RSV strain Long (*wt*RSV). On day 4, five mice per group were sacrificed, and RT-qPCR-derived viral titers in lung tissues were determined and expressed as RSV RNA copies **(B)**. The body weight losses of the remaining five mice were monitored daily for seven consecutive days **(C)**. Data are presented as means ± SD, one representation of three repeated experiments. Mock: mice were infected intranasally with PBS. *p < 0.05, **p < 0.01, ***p < 0.001. ns, not significant. LOD, limitation of detection.

To evaluate the replication and attenuation of these viruses *in vivo*, mice were infected i.n. with 5.0×10^5^ PFU of RLΔM2-2, RLΔM2-2112, and *wt*RSV separately (**Figure 4A**). On day 4, five mice per group were sacrificed, and RT-qPCR was employed to analyze the viral titers in lung tissue, expressed as RSV RNA copies per 0.01 g of lung tissue, as previously described (41, 48). The results indicated that RLΔM2–2 and RLΔM2–2112 had lower mean titers per 0.01 g of lung tissue (4.3 log_10_ copies and 5.4 log_10_ copies, respectively) compared to *wt*RSV (5.7 log_10_ copies). The difference between RLΔM2–2112 and *wt*RSV was not statistically significant (*p* > 0.05); however, RLΔM2–2 replicated to significantly lower extent than *wt*RSV in the lungs (*p* < 0.01) (**Figure 4B**). This result was also consistent with an earlier report, mentioned above, which observed similar findings in MEDIΔM2-2-vaccinated mice (30).

Meanwhile, the changes in the body weight of the infected mice were monitored continuously from day 0 to day 7 post-infection. Mice infected with *wt*RSV or RLΔM2–2112 exhibited a peak body weight loss of approximately 20%, whereas those infected with RLΔM2–2 experienced a maximum loss of less than 10% (*p* < 0.001) (**Figure 4C**). These results suggested that RLΔM2–2 exhibited restricted replication and reduced disease severity compared to *wt*RSV and RLΔM2–2112 in mice (**Figure 4B**).

### The immunogenicity of RLΔM2–2 following intranasal vaccination in BALB/c mice

**Figure 5:**
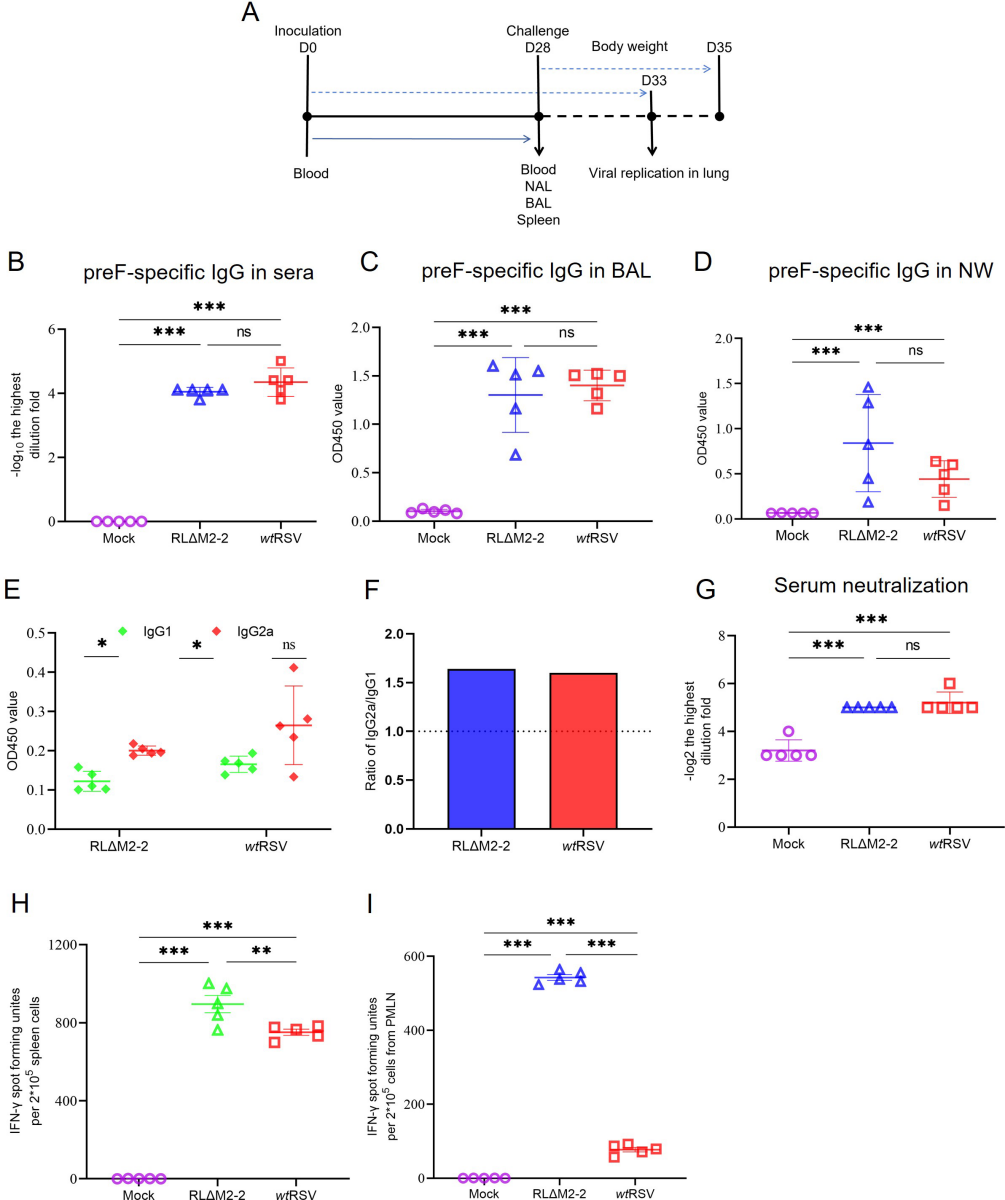
Immunogenicity assessment of RLΔM2–2 in BALB/c mice. Schedule diagram of animal experiments (solid line) **(A)**. Five mice in each group were sacrificed on day 28 post-immunization, and ELISA was performed to detect RSV preF-specific IgG titers in sera **(B)**, BAL **(C)**, and NW **(D)**. Mock: samples were collected from mice inoculated with PBS intranasally. The pre-F-specific IgG1 and IgG2a levels in sera **(E)** and the ratios of IgG2a to IgG1 **(F)** were also measured. Serum-neutralizing antibody levels were assessed by the fluorescent-based neutralization assays (FNA) **(G)**. Mock: sera samples were obtained from mice inoculated with PBS intranasally. RSV M2-1-specific CD8^+^ T-cell immune responses in the spleen **(H)** and in the PMLN **(I)** were evaluated using the ELISpot assay, respectively. Data are presented as means ± SD, one representation of three repeated experiments. *p < 0.05, **p < 0.01, ***p < 0.001. ns: not significant. BAL, bronchoalveolar lavage fluids; NW, nasal washes; *wt*RSV, RSV strain Long; FNA, fluorescent-based neutralization assays; PMLN, pulmonary mediastinal lymph nodes.

Given that RLΔM2–2 exhibited a more favorable attenuation phenotype than RLΔM2-2112, as indicated by lower lung viral replication and reduced body weight loss in BALB/c mice, RLΔM2–2112 was not selected for further evaluation, and subsequent immunogenicity and challenge studies focused on RLΔM2-2. The samples of sera, BAL, and NW were collected from mice 4 weeks after intranasal inoculation (**Figure 5A**). RSV preF-specific serum and mucosal antibodies were detected using ELISA. The ELISA results demonstrated that RLΔM2–2 induced RSV preF-specific IgG in sera (**Figure 5B**), BAL (**Figure 5C**), and NW (**Figure 5D**), comparable to the responses induced by *wt*RSV.

To analyze the Th1/Th2 skewing, preF-specific serum antibodies IgG1 and IgG2a were assessed by ELISA (**Figure 5E**). The ratios of IgG2a/IgG1 of preF-specific serum antibody induced by *wt*RSV and RLΔM2–2 were both greater than 1 (**Figure 5F**), indicating that RLΔM2–2 stimulated Th1-biased immune responses. Neutralizing antibodies incited by RSV F and G play a critical role in preventing RSV disease. Therefore, we tested the induced serum-neutralizing antibody titers using FNA. The results indicated that mice immunized with RLΔM2–2 exhibited neutralizing antibody titers comparable to those induced by *wt*RSV (**Figure 5G**).

Additionally, since the cellular immune responses mediated by CD8^+^ T cell play a protective role in clearing intracellular viruses and reducing disease severity (49), we evaluated the specific CD8^+^ T-cell responses against RSV infection. On day 28 after immunization, lymphocytes were isolated from the spleen and the PMLN and subsequently stimulated with the RSV M2-1-specific H-2K^d^-restricted peptides. The number of IFN-γ-secreting M2-1-specific CD8^+^ T cells was determined using the ELISpot assay. In the spleen, the number of M2-1-specific CD8^+^ T cells secreting IFN-γ was significantly increased in RLΔM2-2-immunized mice compared to both the *wt*RSV group (*p* < 0.01) and the mock group (*p* < 0.001) (**Figure 5H**). Similarly, in the PMLN, the number of IFN-γ-secreting M2-1-specific CD8^+^ T cells was significantly elevated in RLΔM2-2-immunized mice compared to both the *wt*RSV group (*p* < 0.001) and the mock group (*p* < 0.001) (**Figure 5I**). These results indicated that intranasal immunization with RLΔM2–2 induced M2-1-specific CD8^+^ T-cell responses both in the spleen and in the PMLN. We also noted that the difference between RLΔM2–2 and *wt*RSV in the number of IFN-γ-secreting M2-1-specific CD8^+^ T cells was more pronounced in the PMLN than in the spleen. As we know, a key feature of RSV pathogenesis is that it replicates primarily within local mucosal tissues—specifically the superficial epithelial cells—a process that occurs via the luminal surface and is unaccompanied by viremia, thereby largely evading a robust systemic adaptive immune response. Therefore, we deduced that the elevated protein expression from RLΔM2–2 and the resulting presentation by the infected respiratory epithelial cells will directly induce higher CD8+ T-cell responses locally than systemically.

### Protection against *wt*RSV challenge in BALB/c mice immunized with RLΔM2-2 via the intranasal route

**Figure 6:**
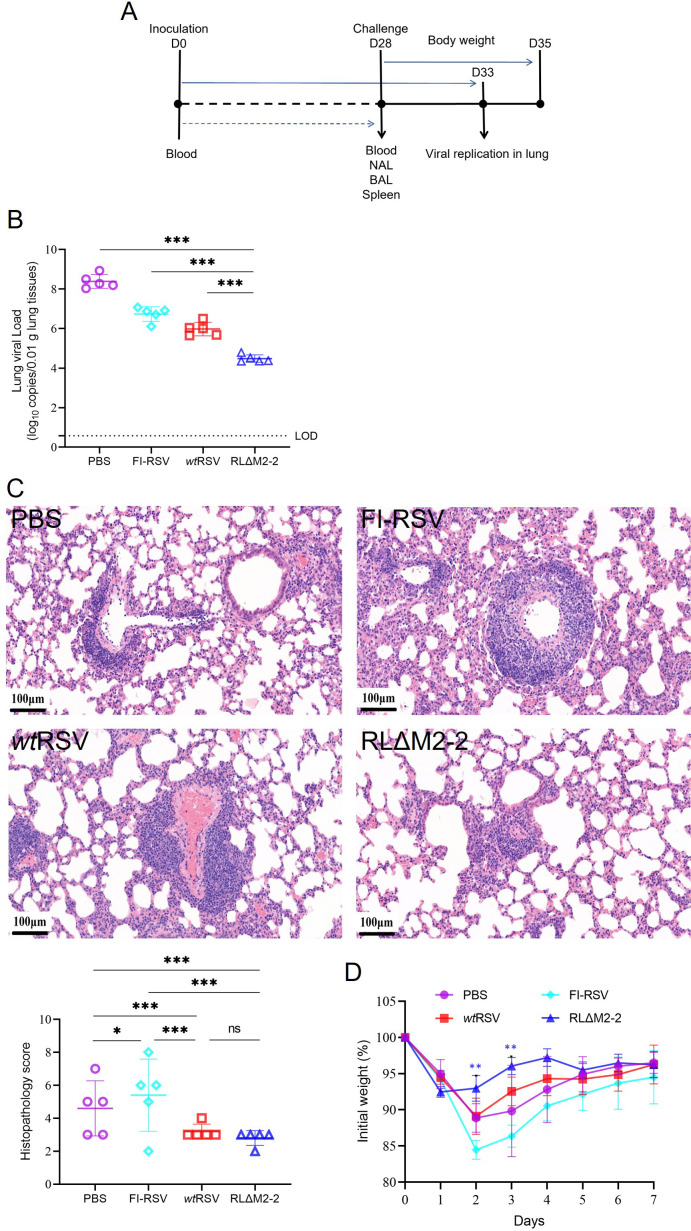
Assessment of immune protection conferred by RLΔM2–2 immunization of BALB/c mice. Schedule diagram of animal experiments (solid line) **(A)**. BALB/c mice were randomly assigned to groups and inoculated intranasally (i.n.) with either 5.0×10^5^ PFU of RLΔM2-2, *wt*RSV, or PBS (50 μl), or intramuscularly (i.m.) with 5.0×10^5^ PFU of FI-RSV. On day 28 post-inoculation, these mice were challenged i.n. with 10^6^ PFU of *wt*RSV. Five days post-challenge, the lungs of immunized BALB/c mice were isolated and evaluated for virus titers by RT-qPCR **(B)** and lung histopathology **(C)**. Meanwhile, the body weights of the same immunized and challenged mice were monitored for seven consecutive days, calculated as a percentage of the initial body weight **(D)**. Data are shown as means ± SD. *p < 0.05, **p < 0.01, ***p < 0.001. ns, not significant. *wt*RSV: RSV strain Long.

To further evaluate the protective immunity induced by RLΔM2–2 against RSV diseases, BALB/c mice were administered i.n. 5.0×10^5^ PFU of RLΔM2-2, *wt*RSV, or PBS. Meanwhile, one group of mice was also administered i.m. with FI-RSV. Four weeks later, these mice were challenged by *wt*RSV with a dose of 10^6^ PFU (**Figure 6A**). Five days post-challenge, viral titers in lung tissues of BALB/c mice were assessed via RT-qPCR. The results indicated that vaccination with RLΔM2–2 significantly decreased viral replication in lung tissue compared to the other groups (*p* < 0.001) (**Figure 6B**).

Subsequently, lung histopathological analyses were conducted on the remaining lung lobe from the same group of mice. FI-RSV was included as a positive control for vaccine-enhanced lung pathology after RSV challenge. Lung tissue lesions were scored using a five-grade scale to assess parameters including thickening of alveolar septa, interstitial pneumonia, and perivascular inflammatory cell infiltration. The RLΔM2–2 group exhibited significantly fewer interstitial inflammatory lesions, characterized by mild perivascular inflammatory cell infiltration and less pronounced alveolar septa thickening. Notably, this group demonstrated the lowest average score of 3.0, which was significantly lower than those of the PBS control group (4.6, *p* < 0.001) and the FI-RSV enhanced-disease group (5.4, *p* < 0.001). Conversely, the score for the RLΔM2-2 group was comparable to that of the wtRSV group (3.2, p > 0.05) (**Figure 6C**). Meanwhile, FI-RSV-immunized mice recapitulated the most severe lung inflammation, consistent with ERD manifestation. In sharp contrast, mice in the RLM2-2 showed no signs of ERD, demonstrating that this live-attenuated candidate circumvents the safety hurdles historically associated with inactivated formulations.

Meanwhile, the changes in body weight of these vaccinated mice following *wt*RSV challenge were monitored over seven consecutive days. Two days post-challenge, mice inoculated with FI-RSV exhibited the most significant weight loss, exceeding 15% of their initial body weight and recovering much slowly. The PBS and *wt*RSV groups also experienced weight loss greater than 10%, although they recovered quickly in the following days. In contrast, RLΔM2-2-immunized mice showed only a minor body weight reduction of approximately 5%, which lasted for only a single day post-challenge and was followed by an immediate recovery in the subsequent days, particularly on days 2 and 3. Consequently, this group exhibited significantly less weight loss compared with both the PBS group (*p* < 0.01) and the FI-RSV group (*p* < 0.01) (**Figure 6D**). Given that, weight loss is a well-established indicator of disease severity, and FI-RSV-vaccinated mice presented the most severe RSV disease following RSV challenge. This phenotypic manifestation was fully consistent with the enhanced lung pathology as introduced above.

Taken together, these results indicated that RLΔM2-2-immunized mice exhibited restricted pulmonary viral replication and alleviated lung pathology, and reduced weight loss following challenge with *wt*RSV, thereby conclusively demonstrating the induction of robust protective immunity against RSV disease. Importantly, ERD was not observed in RLΔM2-2-vaccinated mice following challenge, whereas it was clearly evident in mice vaccinated with FI-RSV.

## Discussion

In this study, we developed two recombinant RSVs, RLΔM2–2 and RLΔM2-2112, by deleting the *M2–2* gene—a key regulator of viral replication and transcription. In the case of RLΔM2-2112, an additional 112-nt region downstream of the *SH* gene was also deleted besides five silent mutations introduced into the end of the *SH ORF*. Our data demonstrated that RLΔM2–2 was attenuated *in vitro* and *in vivo* in the murine model while retaining immunogenicity after intranasal immunization. To our knowledge, this is the first study to evaluate *M2-2-*deleted RSV LAV candidates generated in the RSV strain Long background. Importantly, the present study was not intended to demonstrate that strain Long is superior to A2. Rather, our objective is to determine whether the *M2–2* deletion strategy can also be applied to strain Long, a strain that, despite belonging to subgroup A alongside strain A2, exhibits a distinct immunological profile, as mentioned above. The attenuated replication phenotype and preserved immunogenicity of RLΔM2–2 suggest that strain Long may serve as an alternative parental strain for further RSV LAV development.

Previous studies demonstrated that *M2–2* gene deletion enhances transcriptional activity, consequently elevating the expression of viral antigens (such as F and G proteins) pivotal for inducing neutralizing antibody responses (29). Consistent with these findings, Western blot analysis revealed that both RLΔM2–2 and RLΔM2–2112 exhibited significantly higher expression of the F and G proteins compared to the parental *wt*RSV. This enhanced antigen expression is deduced to contribute to the comparable immunogenicity to *wt*RSV despite the attenuation phenotype, as evidenced by similar levels of RSV preF-specific IgG antibodies in sera, NW, and BAL as well as neutralizing antibodies, and the elevated M2-1-specific and H-2K^d^-restricted CD8^+^ T-cell responses in the spleen and in the PMLN observed in RLΔM2-2-vaccinated mice. Notably, the vaccination of RLΔM2–2 induced a Th1-directed immune response, one of the critical biomarkers to avoid the occurrence of ERD, different from the Th2-directed immune responses induced by the traditional FI-RSV vaccines in the murine model (6). Notably, in the present study, we focused on preF-specific antibodies and serum neutralizing antibody titers to evaluate humoral immune responses, because the pre-F protein is a major target of neutralizing antibodies and a key antigen for RSV vaccine development.

The attenuation of RLΔM2–2 was demonstrated in both *in vitro* and *in vivo* models. In HEp-2 cells, RLΔM2–2 exhibited significantly reduced replication compared to the parental strain, and this attenuation was further validated in BALB/c mice, where the viral titers in lung tissues and body weight loss were substantially lower than those observed with *wt*RSV. Importantly, RLΔM2–2 maintained its replication capacity in Vero cells—a cell line approved for vaccine production—underscoring its potential for industrial-scale vaccine manufacturing. Interestingly, RLΔM2–2112 showed less attenuation compared to RLΔM2–2 in BALB/c mice, although the former only lacks a 112-nt region in the non-coding region downstream of the *SH* gene plus five silent mutations at the end of the *SH* ORF. A similar less-attenuated phenotype was also observed in studies of LIDΔM2–2 based on RSV strain A2 (19), which may be attributed to the fact that viruses with smaller genomes, RLΔM2–2112 versus RLΔM2-2, may replicate more efficiently.

The protective efficacy of RLΔM2–2 was validated by its ability to reduce viral loads, prevent weight loss, and mitigate lung pathology following *wt*RSV challenge. Histopathological analysis revealed significantly lower levels of interstitial pneumonia and inflammatory cell infiltration in the lungs of RLΔM2-2-immunized mice compared to control groups, indicating effective immune protection without excessive inflammatory responses. These findings suggest that RLΔM2–2 provided protective immunity while maintaining a favorable balance between attenuation and immunogenicity in this mouse model. Importantly, the phenomenon of ERD was not observed in mice that were challenged with *wt*RSV following vaccination with RLΔM2-2, as opposed to FI-RSV. ERD has long been the most concerning safety issue in the field of RSV vaccines; while traditional FI-RSV and RSV/hMPV mRNA vaccines have previously triggered such issue, some novel RSV vaccine candidates frequently face significant apprehension regarding this risk when administered to RSV-seronegative children (6, 9).

In the context of existing *M2-2*-deleted RSV LAV family, RLΔM2–2 provides an additional Long-based candidate for further evaluation. As we know, MEDIΔM2–2 was the first of RSV LAV candidates generated by deleting the *M2–2* gene in an A2-derived background and showed acceptable safety and immunogenicity in early-phase clinical studies (21, 30). Thereafter, other M2-2-deleted candidates, including RSV/276, LIDΔM2-2, and RGΔM2-2 (13, 19), exhibited differences in replication or attenuation phenotypes in clinical or preclinical models and indicates that both the viral genetic backbone and the specific design of the *M2–2* deletion will affect the attenuation phenotype. In the present study, RLΔM2–2 showed reduced replication *in vitro* and *in vivo* while retaining immunogenicity after intranasal immunization, suggesting that the ΔM2–2 strategy can also be applied to the RSV strain Long background. However, because we did not perform a direct side-by-side comparison with A2-based ΔM2–2 vaccine candidates, our data do not establish that strain Long provides superior attenuation, genetic stability, or immunogenicity over A2. Future comparative studies should be conducted to determine whether the strain Long backbone confers specific advantages for further development.

Although the immunogenicity and attenuation profiles of RLΔM2-2 determined in this proof-of-concept study are highly preliminary, they warrant further investigation in additional evaluation models, including cotton rats, non-human primates, and human airway epithelial (HAE) cells. Meanwhile, it provides the important basis to develop the next LAV by combining another temperature-sensitivity attenuation mutation such as 1030s (13).

This study has several limitations. First, pre-F-specific IgA was detected in both BAL and NW; however, the resulting titers following a single immunization were close to the limit of detection (data not shown). Although these preliminary findings preclude definitive conclusions, they strongly suggest that a booster immunization strategy is warranted to further enhance mucosal immunity, which is critical for preventing RSV infection within the respiratory tract, particularly the upper respiratory tract. Second, considering the fact that the genetic background of the parent and the deletion strategy of the *M2–2* gene will affect the attenuation phenotype of the resulting RSV LAVs, it is reasonable to conduct a head-to-head comparison study with other *M2-2*-deleted RSV LAVs and evaluate the potential advantage of RSV LAVs constructed by strain Long versus strain A2. Third, apart from F neutralizing antigen, the other neutralizing antigen G protein is also expressed by RSV LAVs and will contribute to the induction of neutralization antibody; therefore, it is also important to analyze G-specific antibody responses in future investigation.

In summary, this study provides proof of concept demonstrating that the *M2–2* deletion strategy can be applied to the RSV strain Long background. The attenuation, immunogenicity, and protective efficacy observed in a murine model support further preclinical evaluation of RLΔM2–2 and its derivates.

## Conclusion

In this study, we successfully engineered two recombinant RSV variants, RLΔM2–2 and RLΔM2-2112, by deleting the *M2–2* gene alone or in combination with the a 112-nt region deletion downstream UTR of the *SH* gene alongside five silent mutations at the end ofthe *SH ORF*. Among these candidates, RLΔM2–2 showed a favorable attenuation profile while retaining substantial immunogenicity. Immunization with RLΔM2–2 induced detectable systemic and mucosal preF-specific antibody responses, serum-neutralizing antibodies, and RSV-specific CD8^+^ T-cell responses with a Th1-skewed immune profile. Importantly, RLΔM2–2 provided effective protection against RSV challenge, significantly reducing lung viral loads and associated pathological damage without inducing ERD.

Together, these findings support RLΔM2–2 as a Long-based RSV LAV candidate that warrants further preclinical evaluation in more permissive animal and human airway epithelium models. Direct comparison with A2-based *M2-2-*deleted candidates will be necessary to determine the relative advantages and limitations of strain Long backbone.

## Data Availability

The whole-genome deep sequencing data presented in this study are deposited in the NCBI Sequence Read Archive (SRA) under BioProject accession number PRJNA1472410. Other data supporting the conclusions of this article are included in the article and its **Supplementary Material**.
