# Supplementary material for: Evaluation of a strain Long human respiratory syncytial virus with M2–2 gene deletion in intranasally vaccinated BALB/c mice
Source: Front Immunol. 2026 Jun 8;17:1829916. doi: 10.3389/fimmu.2026.1829916 (PMC13283998; doi:10.3389/fimmu.2026.1829916)
Supplement: Supplementary file 1 [file Table1.pdf]

Summarizes the nucleotide and amino acid changes identified by deep sequencing of the recombinant RSV strains

To ensure the reliability of variant identification in the deep sequencing analysis of recombinant RSV genomes, we applied stringent filtering criteria. Only single-nucleotide polymorphisms (SNPs) and insertion/deletion mutations (INDELs) with a minimum total sequencing depth (DP) of 500× and at least 10 supporting reads for the alternative allele were considered valid. Moreover, a mutant allele frequency (MAF) threshold of ⩾10% was used to define biologically meaningful mutations, minimizing the impact of sequencing artifacts and enabling the identification of genetically stable or adaptive variants[1-3].

Sequence alignment upon the data from the whole-genome deep sequencing revealed that the leader and trailer regions of the recombinant viruses (RLΔM2-2 and RLΔM2-2112) were completely identical to those of the parental RSV strain Long (GenBank accession number: AY911262). No nucleotide substitutions, insertions, or deletions were detected in all other non-coding regulatory regions including gene starts, gene ends and intergenic sequences with the exception of 112 nts deleted downstream of *SH* gene. In the meanwhile, RLΔM2-2 and RLΔM2-2112 harbored 30 single nucleotide polymorphisms (SNPs) and 24 SNPs, respectively. Among these, the majority were synonymous mutations or missense mutations but leading to conservative amino acid substitutions, indicating minimal impact on viral protein function. Notably, eight and three non-conservative missense mutations were identified within the hypervariable regions of the G protein of RLΔM2-2 and RLΔM2-2112, respectively. Moreover, the amino acid changes in the G protein of RLΔM2-2 accumulated preferentially in the C-terminal third of the protein, which was partial agreement with previous studies[4]. However, no mutations were detected within the highly conserved 13-amino acid core motif in the central region (positions 164 - 176)[4-6], suggesting that these changes did not affect critical structural or functional domains of the protein. No nonsense mutations or amino acid deletions were detected, suggesting that the recombinant viruses maintained overall genomic and proteomic integrity.

This table summarizes the nucleotide and amino acid substitutions identified in recombinant RSV variants by deep sequencing. Amino acid properties were inferred based on hydrophobicity or polarity.

Table 1 Summary of RLΔM2-2 Mutation between parent and progeny viruses

Gene	Nucleotide Change	Amino Acid Change	Mutation Type	AA Property
NS2	T648C	Asp7Asp	Synonymous	-
P	A2386G	Asn14Ser	Missense	Conservative substitution
P	A2417G	Ile24Met	Missense	Conservative substitution
G	A5262G	Lys192Lys	Synonymous	-
G	A5263G	**Lys193Gly**	Missense	Non-conservative substitution
G	A5264G	**Lys193Gly**	Missense	Non-conservative substitution
G	A5265G	**Lys193Gly**	Missense	Non-conservative substitution
G	A5273G	Lys196Arg	Missense	Conservative substitution
G	A5276G	Lys196Arg	Missense	Conservative substitution
G	A5278G	**Thr198Ala**	Missense	Non-conservative substitution
G	A5296G	**Lys204Gly**	Missense	Non-conservative substitution
G	A5297G	**Lys204Gly**	Missense	Non-conservative substitution
G	A5298G	**Lys204Gly**	Missense	Non-conservative substitution
G	A5301G	Lys205Lys	Synonymous	-
G	A5311G	**Lys208Gly**	Missense	Non-conservative substitution
G	A5312G	**Lys208Gly**	Missense	Non-conservative substitution
G	A5317G	**Thr211Ala**	Missense	Non-conservative substitution
G	A5322G	Lys212Lys	Synonymous	-
G	A5323G	Lys213Glu	Missense	Non-conservative substitution
G	A5349G	Lys221Lys	Synonymous	-
G	A5352G	Pro222Pro	Synonymous	-
G	A5354G	Lys223Arg	Missense	Conservative substitution
G	A5361G	Val224Val	Synonymous	-
G	A5382G	Glu232Glu	Synonymous	-
G	A5406G	Lys239Lys	Synonymous	-
G	A5407G	Thr240Ala	Missense	Non-conservative substitution
G	A5411G	Asn242Ser	Missense	Conservative substitution
G	A5418G	Thr244Thr	Synonymous	-
G	A5419G	Thr245Ala	Missense	Non-conservative substitution
L	T8706C	Ile139Ile	Synonymous	-

Table 2 Summary of RLΔM2-2112 Mutation between parent and progeny viruses

Gene	Nucleotide Change	Amino Acid Change	Mutation Type	AA Property
G	A4606G	Lys11Arg	Missense	Conservative substitution
G	A4613G	Leu13Leu	Synonymous	-
G	A4618G	Lys15Arg	Missense	Conservative substitution
G	A5008G	Lys145Arg	Missense	Conservative substitution
G	A5021G	Lys149Lys	Synonymous	-
G	A5031G	Asn153Asp	Missense	Conservative substitution
G	A5035G	Lys154Arg	Missense	Conservative substitution
G	A5036G	Lys154Arg	Missense	Conservative substitution
G	A5044G	Asn157Ser	Missense	Conservative substitution
G	A5046G	Lys158Gly	Missense	Non-conservative substitution
G	A5047G	Lys158Gly	Missense	Non-conservative substitution
G	A5055G	Asn161Asp	Missense	Conservative substitution
G	A5072G	Glu166Glu	Synonymous	-
G	A5079G	Asn169Asp	Missense	Conservative substitution
G	A5127G	Ile185Val	Missense	Conservative substitution
G	A5133G	Lys187Gly	Missense	Non-conservative substitution
G	A5134G	Lys187Gly	Missense	Non-conservative substitution
G	A5136G	Arg188Gly	Missense	Non-conservative substitution
G	A5139G	Ile189Val	Missense	Conservative substitution
G	A5141G	Ile189Val	Missense	Conservative substitution
G	A5146G	Asn191Ser	Missense	Conservative substitution
G	A5156G	Pro194Pro	Synonymous	-
G	A5161G	Lys196Arg	Missense	Conservative substitution
F	T6195G	Asn516Lys	Missense	Conservative substitution

References

1 Domingo, E. & Holland, J. J. RNA virus mutations and fitness for survival. *Annu Rev Microbiol* **51**, 151-178 (1997). https://doi.org:10.1146/annurev.micro.51.1.151

2 Lauring, A. S. & Andino, R. Quasispecies theory and the behavior of RNA viruses. *PLoS Pathog* **6**, e1001005 (2010). https://doi.org:10.1371/journal.ppat.1001005

3 Grubaugh, N. D. *et al.* An amplicon-based sequencing framework for accurately measuring intrahost virus diversity using PrimalSeq and iVar. *Genome Biol* **20**, 8 (2019). https://doi.org:10.1186/s13059-018-1618-7

4 García, O. *et al.* Evolutionary pattern of human respiratory syncytial virus (subgroup A): cocirculating lineages and correlation of genetic and antigenic changes in the G glycoprotein. *J Virol* **68**, 5448-5459 (1994). https://doi.org:10.1128/jvi.68.9.5448-5459.1994

5 McLellan, J. S., Ray, W. C. & Peeples, M. E. Structure and function of respiratory syncytial virus surface glycoproteins. *Curr Top Microbiol Immunol* **372**, 83-104 (2013). https://doi.org:10.1007/978-3-642-38919-1_4

6 Johnson, P. R., Spriggs, M. K., Olmsted, R. A. & Collins, P. L. The G glycoprotein of human respiratory syncytial viruses of subgroups A and B: extensive sequence divergence between antigenically related proteins. *Proc Natl Acad Sci U S A* **84**, 5625-5629 (1987). https://doi.org:10.1073/pnas.84.16.5625
